# Efficacies of DHA–PPQ and AS/SP in patients with uncomplicated *Plasmodium falciparum* malaria in an area of an unstable seasonal transmission in Sudan

**DOI:** 10.1186/s12936-017-1817-9

**Published:** 2017-04-20

**Authors:** Abdelrahim O. Mohamed, Muzamil M. Abdel Hamid, Omer S. Mohamed, Nuha S. Elkando, Abdelmaroof Suliman, Mariam A. Adam, Fahad Awad Ali Elnour, Elfatih M. Malik

**Affiliations:** 1 Department of Biochemistry, Faculty of Medicine, University of Khartoum, Khartoum, Sudan; 2 Neelain Institute for Medical Research, Alneelain University, Khartoum, Sudan; 3 Institute of Endemic Diseases, University of Khartoum, Khartoum, Sudan; 4 Alzohor Health Centre, Damazin, Sudan; 5 Alsalam Health Centre, Damazin, Sudan; 6 Communicable & Non Communicable Diseases Control Directorate, Federal Ministry of Health, Khartoum, Sudan; 7 Department of Community Medicine, Faculty of Medicine, University of Khartoum, Khartoum, Sudan

**Keywords:** Malaria, Dihydroartemisinin–piperaquine, Artesunate, Sulfadoxine–pyrimethamine, Sudan, Genotyping

## Abstract

**Background:**

Artemisinin-based combination therapy (ACT), together with other control measures, have reduced the burden of falciparum malaria in sub-Saharan countries, including Sudan. Sudan adopted ACT in 2004 with a remarkable reduction in mortality due to falciparum malaria. However, emergence of resistance to the first-line treatment artesunate and sulfadoxine/pyrimethamine (AS/SP) has created new challenges to the control of malaria in Sudan. A search for an alternative drug of choice for treating uncomplicated malaria has become inevitable. The objective of this study was to evaluate the therapeutic efficacies of dihydroartemisinin/piperaquine (DHA–PPQ) and AS/SP in an area of unstable transmission in Blue Nile State, Sudan in 2015–16.

**Methods:**

A total of 148 patients with uncomplicated malaria were recruited in the study from November 2015 to end of January 2016. Seventy-five patients received DHA–PPQ while 73 received AS/SP. Patients were monitored for clinical and parasitological outcomes following the standard WHO protocol for a period of 42 days for DHA–PPQ and 28 days for AS/SP; nested PCR (nPCR) was performed to confirm parasite re-appearance from day 7 onwards.

**Results:**

Fifty-five patients completed the DHA–PPQ arm protocol with success cure rate of 98.2% (95% CI 90.3–100%) and one late clinical failure 1.8% (95% CI 0.0–9.7%). The AS/SP showed adequate clinical and parasitological response (ACPR) of 83.6% (95% CI 71.9–91.8%), early treatment failure was 1.6% (95% CI 0.0–8.8%) and late parasitological failure (LPF) was 14.8% (95% CI 7–26.2%). The respective PCR uncorrected LPF was 20%.

**Conclusion:**

DHA–PPQ is an efficacious ACT and candidate for replacement of first-line treatment in Sudan while AS/SP showed high treatment failure rate and must be replaced.

**Electronic supplementary material:**

The online version of this article (doi:10.1186/s12936-017-1817-9) contains supplementary material, which is available to authorized users.

## Background

Malaria is endemic in Sudan and creates a burden to the health system. It has been reported that 7.5 million cases and 35,000 deaths per year were recorded before 2005 [1]. However, national efforts supported by WHO and partners have reduced the burden to one million cases in 2011 and 75% reduction of deaths [2]. Transmission is seasonal and unstable in most parts of the country following rainfall from May to October with peak transmission June to November [3].

Sudan adopted artemisinin-based combination therapy (ACT) in 2004 with artesunate and sulfadoxine/pyrimethamine (AS/SP) as the first-line of treatment, and artemether/lumefantrine (AL) as second-line [4]. SP continued to be used for prophylaxis in pregnancy. Reports from Sudan and other countries have indicated that there is resistance to the antifolate drugs sulfadoxine and pyrimethamine by in vivo efficacy studies and genotyping [5–7]. However, the initial trials of AS/SP justified its selection as the first-line treatment [8]. Successive in vivo trials, following the adoption of AS/SP, continued to show acceptable outcome from different parts of the country, including South Sudan [9, 10].

Dihydroartemisinin/piperaquine (DHA–PPQ) combination has been used in Southeast Asia and other countries in Africa with profound results. DHA is very effective in removal of the asexual stages of *Plasmodium falciparum* together with a gametocidal effect. However, it has a short half-life of 50–60 min [4, 11]. Its partner drug, piperaquine has a long half-life and is effective against asexual malaria parasite stages. Piperaquine has the advantage of not having been used as a monotherapy, except in China in the 1970s [4, 12]. This combination has been reported to provide longer prophylaxis from re-infection after treatment up to 65 days.

With reports of failure and reduced efficacy of AS/SP, especially in eastern Sudan [13], Sudan is seeking an alternative first-line efficacious and affordable treatment. Despite being an efficacious second line of treatment, AL has the disadvantage of being expensive [11]. AL will continue to be the second-line. DHA–PPQ, when found effective, will be a candidate first-line treatment.

## Methods

### Study design

This was an open-label, clinical trial according to WHO guidelines adopted by the National Federal Ministry of Health, Sudan. It is a non-randomized in vivo efficacy clinical trial conducted in two health centres in Dazamin. There was no intention of direct comparison of the patients and treatment outcomes of the two health centres.

### Study area

This study was conducted in two health centres (Alzohor and Alsalam) in Damazin, the capital town of the Blue Nile State in southeastern Sudan. Its population is 281,403, while the population of the entire State is over one million. Rainfall is seasonal from June to October (average rainfall 558.5 ml per year) and malaria transmission follows this season. However Damazin has a second transmission season in December to February, similar to that reported from another area in eastern Sudan [14].

Seventy-five patients were studied for DHA–PPQ and 73 were studied for AS/SP combination therapy according to WHO guidelines [11] during the period from November 2015 to the end of January 2016. According to the protocol, patients presenting with fever or history of fever in the last 24 h and having a positive blood smear for asexual falciparum malaria were candidates for inclusion, after a written consent by patient or a guardian for children. A patient having asexual parasite count more than 1000 and not exceeding 100,000, would be given DHA–PPQ in clinic 1 and AS/SP in clinic 2 according to his/her weight, for three days. The administration of treatment was supervised in the clinics. Blood smears were collected and examined on days 2, 3, 7, 14, 21, 28, 35, and 42 for DHA–PPQ and up to day 28 for AS/SP. Thick and thin blood smears were stained by Giemsa according to standard protocol [15]. Parasite identification and count were performed by a skilled microscopist and subsequently revised by an independent expert microscopist. Where readings disagreed, the reading of the expert was considered final.

Patients were followed up in the above-stated days, when temperature and adverse events of treatment were recorded If parasites were not cleared or symptoms existed, a rescue treatment with AL combination of artemether and lumefantrine was administered.

The data from patient sheets were entered in a predesigned Excel sheet including gender, age, temperature, parasite counts until day 42 for DHA–PPQ and day 28 for AS/SP. Treatment outcomes were based on parasitological and clinical responses according the WHO guidelines [11] and were recorded as adequate clinical and parasitological response (ACPR), early treatment failure (ETF), late parasitological failure (LPF), withdrawal of patient (W) or lost for follow-up (LFU).

### Genotyping of *Plasmodium falciparum msp1* and *msp2* genes

Blood from finger pricks was collected on filter paper (FTA^®^ Whatman paper) on day 0 and the follow-up days, dried and stored in sealed plastic bags. Samples with positive asexual parasitaemia on/or after the seventh day were subjected for molecular analysis. Briefly, DNA was extracted from day 0 and the respective days of re-appearance of parasites using the QIAamp^®^ DNA Mini Kit (Qiagen, Hilden, Germany) for dried blood according to manufacturer protocol. Nested PCR (nPCR) was performed for identification of *P. falciparum* MSP1 and MSP2 allele variants, as described previously [16]. The results were indicated as recrudescence, re-infection or unknown for two lost samples according to WHO guidelines [17].

### Ethical statement

Ethical approval for the study was obtained from the National Research Ethics Review Committee, Federal Ministry of Health, Sudan and a permission to conduct the research was obtained from the Blue Nile State Ministry of Health. Written informed consent was obtained from patients or guardians of minor patients prior to enrollment. Anonymity and confidentiality of patients’ information were maintained throughout this study.

### Data analysis

Data were analysed by Kaplan–Meier analysis using a WHO developed Excel sheet that included the classification of the treatment outcome according to WHO definition, with and without PCR [18].

## Results

A total of 148 patients with uncomplicated falciparum malaria were enrolled in the study, of which 75 received DHA–PPQ and 73 received AS/SP in the two health centres Alzohor and Alsalam, respectively. The mean age of the patients who received DHA–PPQ was 10.8 years and for those who received AS/SP 8.3 years, ranging from one-20 years. The characteristics of the patients of both groups at day 0 are shown in Table 1. The response to DHA–PPQ was adequate. The drug was well tolerated and no adverse events were recorded.

**Table 1:** Characteristics of the study patients in two centres in Damazin, Sudan

	DHA–PPQ patients	AS/SP patients
Age years (range)	10.8 (1–50)	8.3 (1–20)
<5	22	11
5–15	39	57
>15	14	5
Weight kg (SD)	28.6 (17)	23.8 (11.1)
Temperature °C (range)	36.9 (35–38)	37.3 (36.6–38)
Parasite Gmean (range)	23,007 (1000–100,000)	9611 (1600–100,000)
Gametocytes	7 (9.6%)	7 (10%)

On follow-up (Table 2) with PCR uncorrected, only those patients who were present at day 3 were included in the follow-up: DHA–PPQ was 73 patients and AS/SP was 70 patients. Fifty-four patients had ACPR while 18 patients were lost for follow-up of DHA–PPQ. One patient had LCF. The corresponding figures of the AS/SP are 51 ACPR, one ETF and 13 LPF; five patients were lost to follow-up (Table 2).

**Table 2:** PCR uncorrected follow-up results of the patients

Outcome	DHA–PPQ N (%) (95% CI)	AS/SP N (%) (95% CI)
ETF	0 (0%) (0.0–6.5)	1 (1.5%) (0.0–8.3)
LCF	1 (1.8%) (0.0–9.7)	0 (0%) (0.0–5.5)
LPF	0 (0%) (0.0–6.5)	13 (20%) (11.1–31.8)
ACPR	54 (98.2%) (90.3–100)	51 (78.5%) (66.5–87.7)
Total	55	65

Gametocytes were found in seven patients (9.6%) in the DHA–PPQ centre and seven (10%) in the AS/SP centre. During follow-up no gametocytes were recorded in DHA–PPQ group while four (5.7%) patients from the AS/SP centre had gametocytes.

**Fig. 1:**
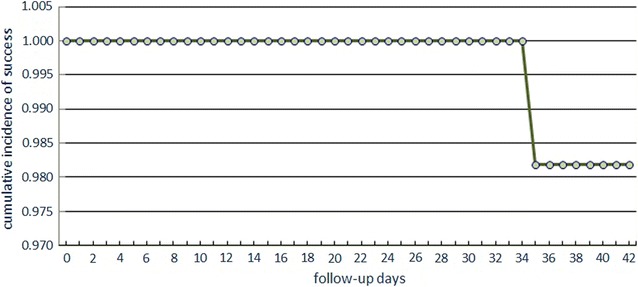
Kaplan–Meier survival curve for DHA–PPQ treatment (PCR corrected)

**Fig. 2:**
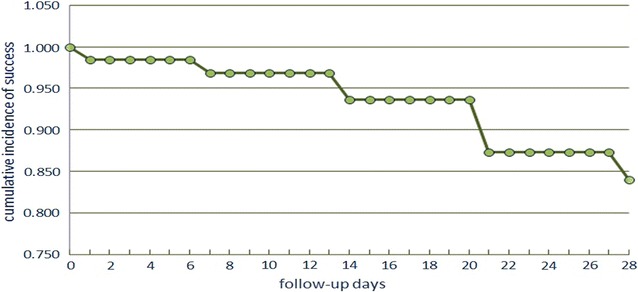
Kaplan–Meier survival curve for AS/SP treatment (PCR corrected)

Table 3; Figs. 1 and 2 show the PCR-corrected respective numbers for DHA–PPQ and AS/SP. Kaplan–Meier survival curves in Figs. 1 and 2 are representation of the results in Table 3. The one patient denoted as LCF in DHA–PPQ at day 35 was a recrudescence, while nine of the AS/SP group were recrudescence, two were re-infection, and two samples were lost.

**Table 3:** PCR corrected follow-up results of the patients

Outcome	DHA–PPQ (N%) (95% CI)	AS/SP (N%) (95% CI)
ETF	0 (0%) (0.0–6.5)	1 (1.6%) (0.0–8.8)
LCF	1 (1.8%) (0.0–9.7)	0 (0%) (0.0–5.9)
LPF	0 (0%) (0.0–6.5)	9 (14.8%) (7–26.2)
ACPR	54 (98.2%) (90.3–100)	51 (83.6%) (71.9–91.8)
Total	55	61

## Discussion

This efficacy study has shown that DHA–PPQ is effective in the treatment of uncomplicated falciparum malaria in an area of unstable transmission in Sudan. The single late parasitological failure on day 35 proved to be a recrudescence after verification by nPCR. The response to the treatment was dramatic and all slides were negative from day 1 and continued to be so to the end of follow-up. There were no complaints of adverse complications due to the treatment. There were no severe adverse events recorded during the follow up. As this is the first time the drug has been tried in Sudan there are no previous reports for comparison.

Other research in neighbouring countries has shown similar effectiveness [19, 20]. In Kenya, a study of efficacy of AL and DHA–PPQ in children under 5 years of age showed that DHA–PPQ is an effective and tolerable treatment for uncomplicated malaria. DHA–PQQ was adopted as a second-line treatment in Kenya in 2009 [19]. Equally, research in west, south and central Africa has shown similar results [20–22]. Dihydroartemisinin has been identified as a potent derivative of the parent drug artemether [4]. Its partner drug, piperaquine, has only been used as monotherapy for treatment of malaria in the 1970s in China and has never been used in Sudan or any other African country [12, 23]. However, the combination DHA–PPQ has only been used in Sudan lately in research and is being used in other African countries. This will make DHA–PPQ an ideal ACT for malaria in Sudan.

On the other hand, AS/SP has lost its effectiveness against falciparum malaria in Blue Nile region. Besides the single case ETF, 20% of the patients showed late parasitological failure and four patients had gametocytes on days 21 and 28 of the follow-up (Table 2). The corrected PCR results have shown that the failure rate was more than 14%, which is alarmingly high. This finding indicates that AS/SP has lost its position as an effective treatment for falciparum malaria in the Blue Nile region, similar to other reports from Kassala (15%) and Gedaref (14%) [13]. In fact, patients and medical personnel have started to lose confidence in this combination, and are using instead quinine and injectable artemether. The partner drug, SP was used before 2005 as a second-line treatment for falciparum malaria in Sudan [24]. It has been known to exhibit resistance before adoption of ACT in 2004 [8, 9, 24]. SP is also used alone as prophylaxis in pregnancy. AS/SP itself is not a fixed combination as it co-exists in separate compartments in the blister seal. All these factors led to an increase of resistance of the parasite to SP, which is responsible for ECF and LPF, especially LPF, as SP is responsible for long-term clearance of parasites [4, 11]. There were also remaining gametocytes in the late stages of follow-up in this study. Artesunate is known to have gametocidal effect [11]. However, those subjects with gametocytes remain infective to *Anopheles* mosquitoes and thus raise a question of potency of artesunate as a gametocidal therapy. The success of malaria control in Sudan largely depends on the use of AS/SP and AL in the treatment of cases beside other control measures. Failure of any of the control measures, especially treatment, will jeopardize that success. The health authority has acknowledged the failure of AS/SP and the need for change with DHA–PPQ.

## Conclusion

Sudan is on its way to changing its first-line malaria treatment and the proposed DHA–PPQ is an ideal alternative. The policy makers at the Federal Ministry of Health have decided to choose AL as first-line malaria treatment and DHA–PPQ as a second-line malaria treatment. A decree has been issued to this effect by the Federal Minister of Health, Sudan on March 9th, 2017. This study has shown that DHA–PPQ would suitable as a first-line treatment in an area of unstable transmission in Sudan, but the Ministry of Health has chosen it as second-line. AS/SP has lost its place as first-line therapy for uncomplicated falciparum malaria and the results of this study justify its replacement.

## Additional files

**Additional file 1.** DHA-PPQ treatment group.

**Additional file 2.** AS-SP treatment group.

## Abbreviations

ACT: artemisinin-based combination therapy
AS/SP: artesunate–sulfadoxine–pyrimethamine
DHA–PPQ: dihydroartemisinin–piperaquine
AL: artemether–lumefantrine
SP: sulfadoxine–pyrimethamine
WHO: World Health Organization
nPCR: nested polymerase chain reaction
ACPR: adequate clinical and parasitological response
ETF: early treatment failure
LPF: late parasitological failure
LCF: late clinical failure
W: withdrawal of patient
LFU: lost for follow-up
MSP: merozoite surface protein
G mean: geometric mean
